# Molecular Determinants of Epidermal Growth Factor Binding: A Molecular Dynamics Study

**DOI:** 10.1371/journal.pone.0054136

**Published:** 2013-01-24

**Authors:** Jeffrey M. Sanders, Matthew E. Wampole, Mathew L. Thakur, Eric Wickstrom

**Affiliations:** 1 Department of Biochemistry and Molecular Biology, Thomas Jefferson University, Philadelphia, Pennsylvania, United States of America; 2 Department of Radiology, Thomas Jefferson Medical College, Philadelphia, Pennsylvania, United States of America; 3 Kimmel Cancer Center, Thomas Jefferson University, Philadelphia, Pennsylvania, United States of America; Monash University, Australia

## Abstract

The epidermal growth factor receptor (EGFR) is a member of the receptor tyrosine kinase family that plays a role in multiple cellular processes. Activation of EGFR requires binding of a ligand on the extracellular domain to promote conformational changes leading to dimerization and transphosphorylation of intracellular kinase domains. Seven ligands are known to bind EGFR with affinities ranging from sub-nanomolar to near micromolar dissociation constants. In the case of EGFR, distinct conformational states assumed upon binding a ligand is thought to be a determining factor in activation of a downstream signaling network. Previous biochemical studies suggest the existence of both low affinity and high affinity EGFR ligands. While these studies have identified functional effects of ligand binding, high-resolution structural data are lacking. To gain a better understanding of the molecular basis of EGFR binding affinities, we docked each EGFR ligand to the putative active state extracellular domain dimer and 25.0 ns molecular dynamics simulations were performed. MM-PBSA/GBSA are efficient computational approaches to approximate free energies of protein-protein interactions and decompose the free energy at the amino acid level. We applied these methods to the last 6.0 ns of each ligand-receptor simulation. MM-PBSA calculations were able to successfully rank all seven of the EGFR ligands based on the two affinity classes: EGF>HB-EGF>TGF-α>BTC>EPR>EPG>AR. Results from energy decomposition identified several interactions that are common among binding ligands. These findings reveal that while several residues are conserved among the EGFR ligand family, no single set of residues determines the affinity class. Instead we found heterogeneous sets of interactions that were driven primarily by electrostatic and Van der Waals forces. These results not only illustrate the complexity of EGFR dynamics but also pave the way for structure-based design of therapeutics targeting EGF ligands or the receptor itself.

## Introduction

Receptor tyrosine kinases (RTK) play essential roles in numerous cellular processes. Activation of an RTK by a particular ligand(s) enables transduction of a biological signal from the membrane surface to intracellular signaling pathways [1]. Ligand binding to the extracellular domain of an RTK promotes dimerization, leading to auto-phosphorylation by the intracellular kinase domain [2]. One subgroup of the RTK family, the ErbB or Her family, includes the epidermal growth factor receptor (EGFR, ErbB1, Her1). EGFR is necessary for cell proliferation and survival. Misregulation of the ErbB family, either through ErbB ligands or the receptors themselves, has been implicated in several diseases including glioblastoma, breast, skin, and lung cancer [3].

**Figure 1:**
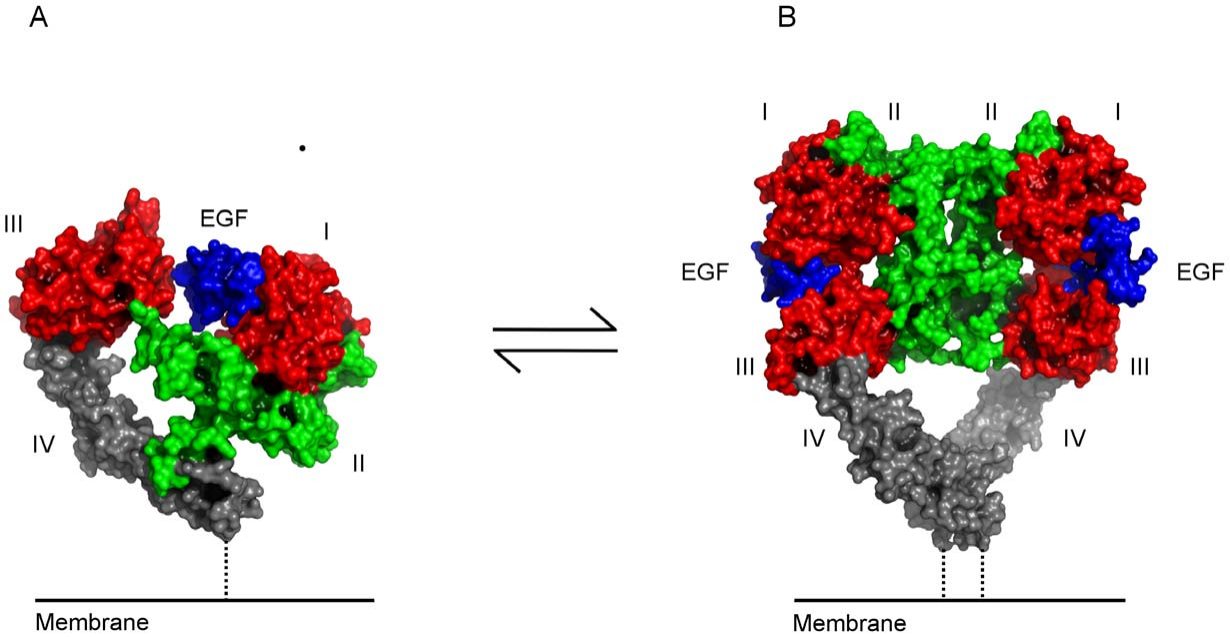
Structural model of ligand dependent activation of EGFR. a) Structure of EGF (blue) bound to domain I of the auto-inhibited conformation of the extracellular domain. The two ligand binding domains are colored red, domain II green and domain IV grey. b) Upon ligand binding EGFR coordinate the two ligand binding domains in a clamp like fashion and dimerization occurs.

As with all RTKs, activating ligands bind to the extracellular domain of EGFR. The mechanism of ligand-dependent activation of EGFR has been studied in great detail [4]. High-resolution crystal structures of the extracellular domain of EGFR in the ligand-bound and unbound states demonstrated that binding of EGF promotes several large-scale conformational changes leading to EGFR dimerization (Figure 1) [5], [6]. These studies also showed that EGFR ligand binding is bivalent. Two beta-solenoid domains of EGFR clamp EGF in the ligand binding site, while two cysteine-rich domains control auto-inhibition by burying the dimerization interface in the absence of a ligand.

Seven different ligands are known to bind to EGFR: epidermal growth factor (EGF), betacellulin (BTC), heparin-binding EGF-like growth factor (HB-EGF), amphiregulin (AR), epiregulin (EPR), transforming growth factor-α (TGF-α), and epigen (EPG) [7]. They are synthesized as transmembrane proteins that are cleaved to produce soluble growth factors. Each soluble EGFR ligand shares a common fold termed the EGF-like motif. This fold is characterized by a consensus sequence of spatially conversed cysteine residues that form three disulfide bonds. Additionally, HB-EGF and AR contain amino-terminal heparin binding domains. Structural analysis of six of these ligands, with EPG being the exception, illustrated a common globular structure [5], [8]–[10].

While much attention has been paid to the biochemical and physiological roles of EGFR ligands, little is known about the protein-protein interactions that determine the binding affinity of a given ErbB ligand. EGFR ligands generally fall into two classes: high affinity or low affinity. High affinity ligands (EGF, TGF-α, HB-EGF, and BTC) bind with a dissociation constant (K_d_) between 1 and 100 nM, while low affinity ligands (AR, EPR, and EPG) show a K_d_ greater than 100 nM [11], [12]. Ranking of these ligands has been difficult as previous reports have used a variety of binding conditions, receptor constructs, and cell lines. Some studies have not controlled for heterodimerization of EGFR with another ErbB ligand, or ligand binding to another ErbB receptor. Beyond knowledge of the ranking of the seven ligands, understanding the molecular determinants of EGFR ligand binding may provide insight into the observations that differences in cellular signaling by EGFR occur when cells are treated with different agonists or different concentrations of the same ligand for a particular ErbB receptor [13].

In this study we explore the interactions between the extracellular domain of EGFR and its ligands in order to understand the side chain and backbone interactions that give rise to the experimentally observed binding affinities. While biophysical analyses can provide information on intermolecular interactions, molecular dynamics (MD) simulations provide atom-by-atom resolution and dynamical behavior on a nanosecond timescale. Previous modeling studies of EGFR have illustrated the asymmetric nature of dimer formation and kinase function. Using theoretical free energy methods combined with conventional MD simulations, we sought to determine residue-residue interactions that give rise to binding affinities for each EGFR ligand.

## Results

### Docking and molecular dynamics simulations of EGFR ligands

To explore the differences in binding affinity of an EGFR ligand to the receptor, we chose to use computational methods to dock each ligand to the extracellular domain of EGFR (sEGFR) and compute the relative free energies. Accurate free energy prediction of protein-protein interactions is one of the long standing goals of computational biology [14]. Due to high computational costs, accuracy of force field parameters, and complexity of large solvated systems, computational free energy studies can be time consuming and may only yield approximate binding affinities. With these limitations in mind, we chose to simulate the sEGFR dimer bound to each ligand and compute only relative free energy differences. This allows us to rank each ligand but prevents determination of the absolute binding energy. Computation of absolute binding energies comes with a high computational cost for each system (>200 k atoms), a quasi-harmonic approximation of entropy, and is further complicated by energetic contributions from transmembrane and intracellular domain association in the full length EGFR receptor.

**Figure 2:**
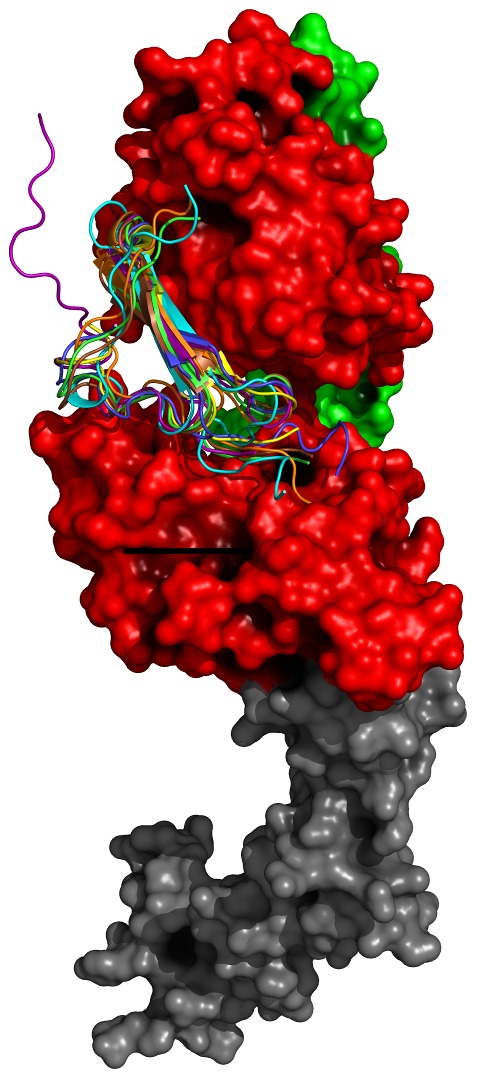
Docking poses of EGFR ligands. Using the EGFR dimer bound to EGF as a starting structure, the remaining six ligands were docked to the binding pocket by alignment to the backbone of EGF (Blue). AR is colored purple, BTC cyan, EPG brown, EPR green, HB-EGF yellow and TGF-α orange.

The crystal structures for the sEGFR dimer structure bound to two EGF molecules (PDB code: 1IVO) were used as a starting model for the docking of each ligand. In this structure, electron densities for the ligand binding domains (DI and DIII) and the domain containing the dimerization arm (DII) were well resolved. Domain IV, however, was more disordered and initially could not be fit to the density maps. Therefore we chose to model the fourth domain DIV using the inactivated structure of EGFR bound to EGF (PDB code 1NQL) as a reference [15]. A structural alignment of our model compared to the recently resolved x-ray structure of EGF-EGFR (PDB code: 3NJP) revealed an overall root mean squared deviation of 2.89 Å [16]. Another crystal structure of an EGFR dimer has been previously determined bound to TGF-α [5]. Both dimer structures illustrated similar positions of the ligand in the binding pocket and similar conformations of the first three domains (Figure S2). To dock the other five known EGFR ligands, each ligand's backbone was aligned with position-conserved atoms in EGF (Figure 2). To remove any steric overlap, we performed a 1000-step energy minimization using the Amber force field parameters in the molecular visualization program Chimera [17] (Table 1).

**Table 1:** Summary of structures used in MD simulations.

Ligand	Number of residues in structure	PDB code	Method	R.M.S.D. (Å)*	Binding affinity (K_d_)
EGF	47	1IVO	X-ray	0.00	0.6 nM
AR	50	2RNL	NMR	1.45	350 nM
BTC	50	1IPO	NMR	4.00	1.4 nM
EPR	46	1K37	NMR	1.90	2.8 µM***
HB-EGF	40	1XDT	X-ray	0.93	7.1 nM
EPG	42	Q6UW88**	Homology	2.05	>500 nM
TGF-α	50	1MOX	X-ray	3.09	9.2 nM

* : Root mean square deviation relative to EGF in 1IVO structure,

** : SWISS-MODEL repository code for homology model database [41], [42],

*** : Murine Epiregulin.

**Figure 3:**
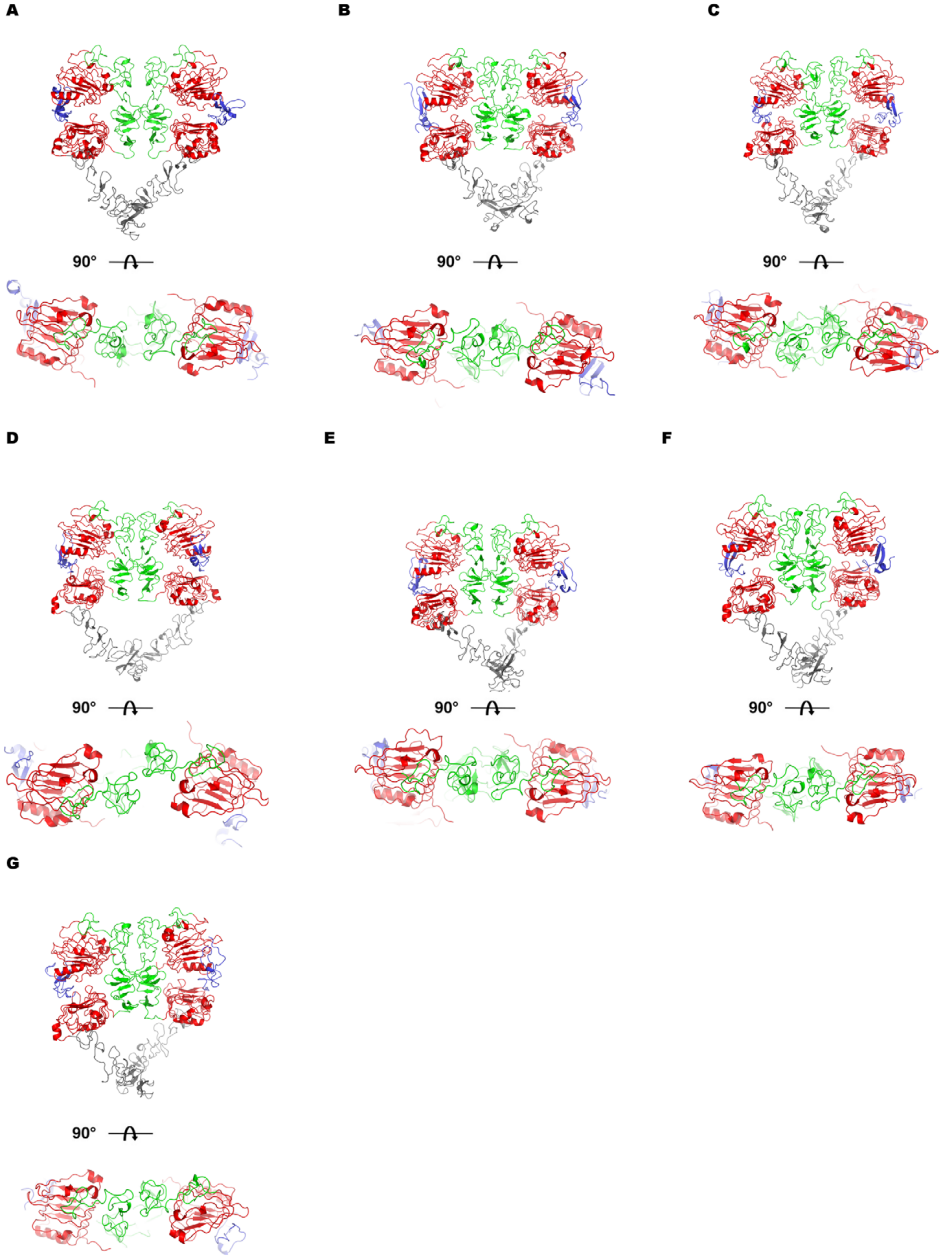
Alteration of the dimerization interface by ligand binding. Structural snapshots were taken at the end of the 25.0 ns production runs. Top views generated by a 90 degree rotation about the x-axis of the structure provide a clear view of the dimerization loops (EGFR residues 190–208) thought to influence the overall strength of the an EGFR-ligand complex. Structures were generated for each ligand: a) AR, b) BTC, c) EGF, d) EPG, e) EPR, f) HB-EGF and g) TGF-alpha.

To determine if each ligand causes different structural alterations in the EGFR dimer, we compared conformations of each ligand-protein complex at the end of the production simulations to the TGF-α-EGFR and EGF-EGFR complexes. A recent structural study of an ErbB4-neuregulin-1β complex highlighted two different types of interactions between ligand-ErbB complexes *in vitro* [18]. In this study the authors identified a rotational motion about the dimerization arms that disrupts the dimer contacts, giving rise to asymmetric dimers. They determined this by studying the orientations of the dimer interface for known ErbB-ligand structures, particularly two coordinated loops (EGFR residues 190–208). In the case of their ErbB4 structure and that of EGF-EGFR, these loops are staggered with respect to each other in the dimer interface while their positions are flush in the TGF-α-EGFR structure (Figure S3). From these observations they concluded that flush dimer interfaces were more stable than staggered interfaces, giving rise to the observed negative cooperativity in cell binding assays. Our simulations revealed both staggered and flush interfaces (Figure 3). In the extreme case, EPG was unable to adopt a flush conformation and the loops were distorted by the rotation about domains I and III. This exposure may partially explain low binding affinity and also agrees with the PBSA/GBSA results discussed below. EGF and TGF-α induced flush conformations while AR, BTC, HB-EGF, and EPR adopted staggered conformations.

### Ligand sequence and structural conversation

**Figure 4:**
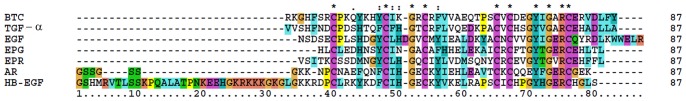
Sequence alignment of the EGFR ligands. a) Shown are the seven ligands used in the computational studies. Sequences of only the EGF like domains of each ligand were used in the alignment. “*” represent 100% conservation while “.” and “:” represent partial sequence conservation.

Each EGFR ligand shares a common EGF-like motif (or fold). This fold is characterized by three intramolecular disulfide bonds constraining three solvent-accessible loops [19]. In addition to the conserved cysteines, several other residues are conserved: two aromatic residues (tyrosine or phenylalanine) at positions 13 and 22 (numbering based on position in EGF sequence), a nonpolar residue at position 15, polar residue at 16, glycine at 18 and 39, tyrosine at position 37, and a conserved arginine at 41 (Figure 4). Mutational studies of tyrosine 13 demonstrated that substitution with the phenylalanine or leucine retains 75% binding affinity relative to wild type while other mutations cause a reduction of more than 90% in binding affinity [20]. In addition, AR and HB-EGF also possess N-terminal heparin binding domains that are known to help stabilize the EGFR dimer with heparin sulfate [21], [22]. An atomic structure of EPG, the last EGFR ligand to be identified, had not been determined at the time of this study. Therefore we used a homology model of EPG based on the homologous ligand EPR (44% sequence homology) [23]. After minimized EGFR-ligand structural models were obtained for all seven ligands, each was subjected to a 25 ns molecular dynamics simulation. Solvent accessible surface area (SASA) values are one measure of system stability SASA plots of each ligand-receptor system were used to tell if the system was stable and converged during the 25 ns simulation (Figure S1). These results indicated that each EGFR dimer-ligand system was stable after 5.0 ns of a molecular dynamics simulation.

### MMPBSA ranking of EGFR ligands

While molecular dynamics simulations can provide us with a qualitative assessment of intramolecular interactions, knowledge of the energetic contributions from individual atoms or residues can provide a quantitative analysis of the binding mode of a ligand to a protein. Molecular mechanics Poison-Boltzmann Surface Area (MM-PBSA) calculations are a computationally efficient method to compute relative binding affinities [24]. Enthalpy terms are computed from the molecular mechanics energies recorded during the simulation and the solvation of the receptor and ligand using continuum solvent models coupled with salt models to account for ionic solvent effects. Entropy is calculated using normal mode analysis and can be is computationally expensive. It has been shown that relative free energies omitting the entropy terms can be used to calculated relative affinities and accurate ranking of ligands [25]. MM-GBSA methods are more frequently used to predict absolute binding energies because MM-PBSA depends more heavily on the internal dielectric constant of the solute, which can vary greatly depending on the system and the number of internal ionizable groups [26]. Since the errors are dependent on sequence content, ligands with homologous sequences would be expected to have similar errors. This dielectric estimation error would not affect PBSA when predicting accurate rankings, but could fail on absolute binding energy predictions. Given the size of each EGFR dimer-ligand complex, we chose to compute only relative binding energies to see if MM-PBSA/GBSA could rank the EGFR ligands based on the high and low affinity states. MM-PBSA results (Table 2) ranked the ligands in the following order: EGF>HB-EGF>TGF-α>BTC>EPR>EPG>AR. MMGBSA produced different ranking results: EGF>TGF-α>HB-EGF>BTC>EPR>AR >EPG. (Table S1). Nonpolar solvation energies contributed favorably in all cases, as did electrostatic and van der Waals forces. The polar contributions to EGFR binding, however, were significantly unfavorable to binding in all complexes. This suggests that the overall driving forces for EGFR ligand binding are favorable van der Waals and electrostatic interactions, with little contribution from nonpolar solvation energies.

**Table 2:** Free energy results from MM-PBSA.

Ligand	ΔE^ele^ *	ΔE^vdw^ **	ΔG^PB^ ***	ΔG^SA^ ****	ΔG^MMPBSA^
EGF	−176.5(8.30)	−148.12(10.48)	239.80(12.67)	−17.42(0.88)	−102.29(5.56)
HB-EGF	−365.82(2.65)	−115.22(8.08)	402.20(13.29)	−13.17(0.71)	−92.30(2.09)
BTC	−425.56(66.03)	−115.78(6.69)	469.92(47.26)	−13.49(0.87)	−84.91(3.00)
TGF-α	−146.22(55.20)	−132.43(2.40)	217.96(42.92)	−15.38(0.12)	−86.08 (0.16)
AR	−207.08(0.87)	−100.29(1.13)	243.34(8.88)	−12.20(0.43)	−66.25(0.16)
EPR	−211.75(34.05)	−117.49(13.06)	265.01(31.06)	−13.77(1.21)	−77.99(12.92)
EPG	−121.95(13.23)	−91.51(11.99)	150.58(12.80)	−13.65(0.34)	−76.14(12.17)

All units are given in kcal/mol. The standard state is taken to be 1 M.

* : ΔE^ele^: coulombic energy.,

** :ΔE^vdw^ : van der Waals energy.

*** :ΔG^PB^: Poisson-Boltzmann polar solvation energy.

**** :ΔG^SA^ :non-polar solvation energy. Standard Errors of corresponding values are given in parentheses.

### Hydrogen bond and salt bridge analysis reveal heterogeneous sets of interactions

**Figure 5:**
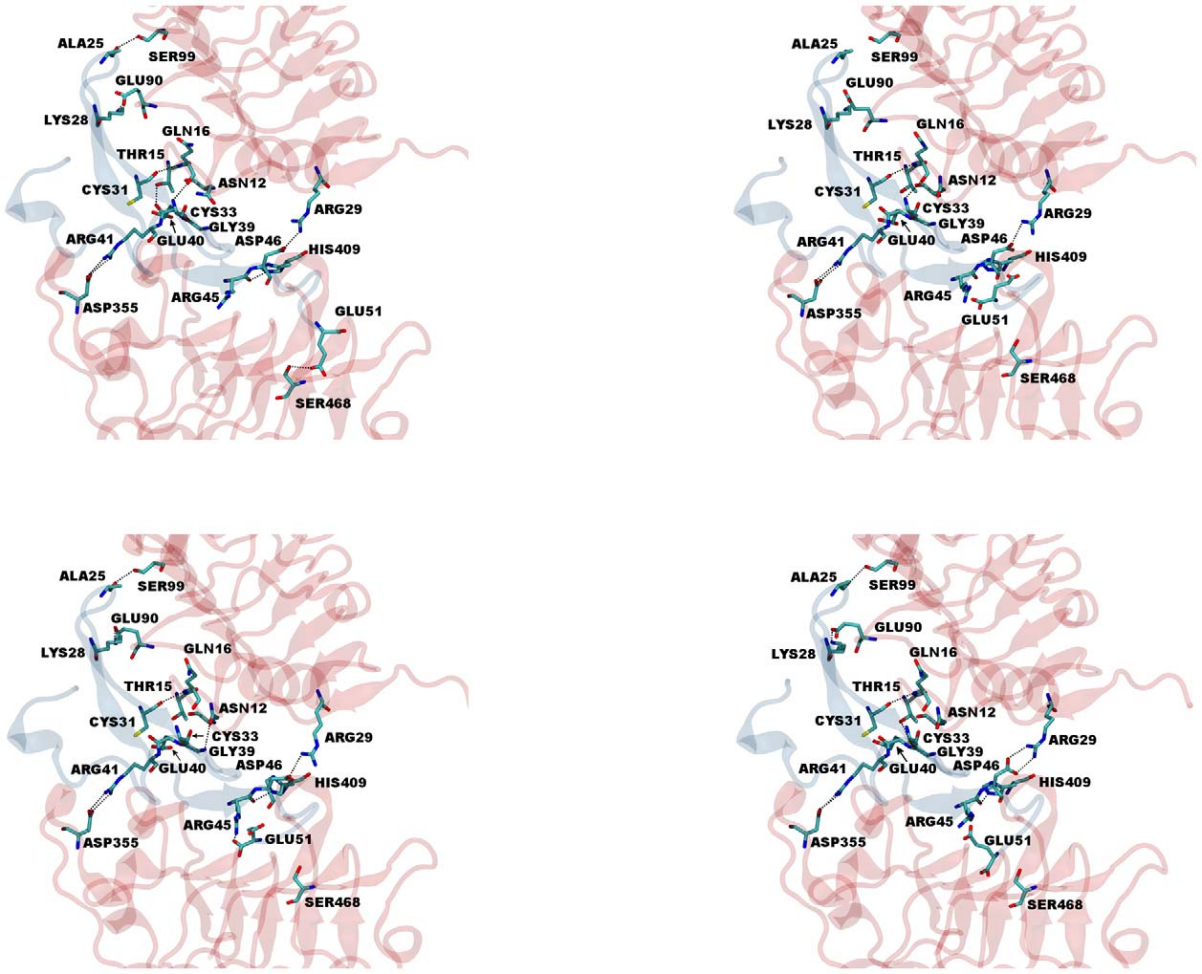
Hydrogen bonding analysis of last 6.0 ns used for energy calculations. Snapshots of the EGFR domains I and III and EGFR protein-protein interfaces from simulations were taken at a) 19.0 ns b) 21.0 ns c) 23.0 ns and d) 25.0 ns. Dotted lines represent hydrogen bonds that had lifetimes of greater than 1.2 ns of the 6.0 ns time frame. The ligand binding domains are represented in red and EGF in blue.

Hydrogen bond lifetimes and salt bridges are indicators of stable non-covalent interactions during the course of a MD simulation. To investigate the interactions between EGFR and a given ligand at the molecular level, we analyzed the last 6.0 ns of each simulation using hydrogen bond and salt bridge analysis. We chose to analyze only the last 6.0 ns as we want to ensure the stability of each system. High occupancy hydrogen bonds were observed for all ligands bound to EGFR (Figure 5). The results, summarized in Table 3, suggest several conserved residues are involved in hydrogen bonding and salt bridge formation. Our expectation was that hydrogen bonding patterns, or lack thereof, would be similar for both the high and low affinity ligand classes. A common set of hydrogen bonds in all simulations occurred between glutamine 16 of EGFR and the backbone of the fourth and fifth conserved cysteines of each ligand. Rather unexpectedly, high and low affinity ligands did not share any additional hydrogen bonding patterns. We did observe that the number of salt bridges (Table 4) decreased in the lower affinity ligands. AR, EPG, and EPR formed only two salt bridges during the 6.0 ns simulation window. This suggests that the ability to form energetically favorable salt bridges is a factor in EGFR ligand affinity.

**Table 3:** Residues involved in hydrogen bonding during the last 6.0 ns of each simulation.

EGFR residue	EGF	AR	BTC	TGF-α	HB-EGF	EPR	EPG
SER11	TYR44						
ASN12	GLY39	GLY44			GLY34		
LYS13	GLU40	GLU45			GLU35		GLU36
LEU14		THR35	CYS32	CYS32	SER25		
THR15	GLU40, CYS31	GLU45			GLU35,CYS26		GLU39
GLN16	CYS33, CYS31,ASN32	CYS38,CYS36, LYS26	CYS32, CYS34	CYS32,CYS34	CYS28, CYS26,LYS16	CYS32, TYR29,CYS30,	CYS27, CYS29, ARG28
LEU17				GLY40			
GLY18							CYS29
ASP22		GLN40					
SER26			GLU36				
ARG29	ASP46		GLU36				
TR45			CYS32				
GLU90	LYS28			LYS29			LYS24
SER99	ALA25	HIS30	THR29	SER3	GLU20	ASP24	
TYR101		HIS30			GLU20		
ARG125			GLU27	GLU27			
SER127			GLU27	GLU27			
ASN128					GLU20		
VAL350				GLU44		GLU42	GLU39
ARG353		ILE21	GLU44	GLU44			GLU39
ASP355	ARG41	ARG46		ARG42	ARG36	ARG40	
SER356		ASN18			ASP8	TYR13	ASP6
GLN384		LYS50	GLY40		HIS12	PHE43, GLU42	LEU41
GLN408					LEU40		
HIS409	ARG45						
SER418			TYR50	ALA46		LEU46	
LYS443			TYR50				
SER468	GLU51,TRP50						
ARG470			TYR50	ALA50			

**Table 4:** Salt bridges formed during 6.0 ns of each simulation.

Salt Bridges							
EGFR residue	EGF	AR	BTC	TGF-α	HB-EGF	EPR	EPG
LYS13	GLU39	GLU45		GLU90	GLU35		GLU36
ASP355	ARG41	ARG46	LYS13, ARG42	ARG42		ARG40	
ARG29	ASP46		GLU36				
ARG353			GLU44	GLU44	ASP8		GLU39
ASP22	LYS48						
ARG125			GLU27	GLU27			
SER418							
LYS443	GLU51						
ASP102			ARG22	ARG22, HIS4			
GLU90	LYS28				ARG22		

### Free energy decomposition of EGFR-ligand complexes

Binding hotspots on protein interfaces can dictate the experimentally observed binding affinity. While alanine scanning mutagenesis can provide some insight, it must be interpreted with caution as exact knowledge of structural perturbations must be considered [27]. Virtual mutagenesis or energy decomposition to minimize the perturbation of the protein-ligand complex by alanine substitution is an attractive alternative. To determine the molecular interactions that give rise to the ranking of the EGFR ligands, residue level decomposition analysis was performed to identify sequence and structural motifs that may be affecting a ligand's binding affinity. To compare our GBSA/PBSA results ranking results, we performed decomposition using both the GB/PB models. We hypothesized that conserved residues in the EGFR binding ligands may be necessary for high affinity binding in addition to protein stability. We were also curious to see if the differences between high and low affinity correlated with a subset of position-specific amino acid residues that force EGFR in to a certain conformational state.

**Figure 6:**
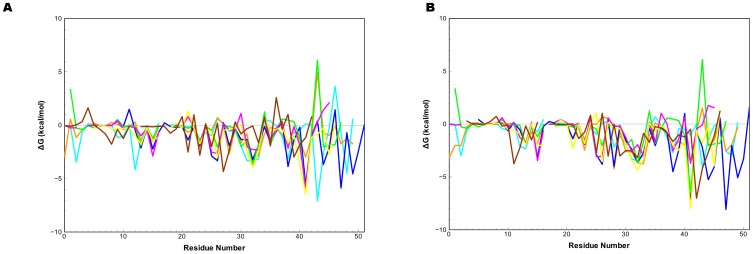
Comparison of decomposition energy values of GBSA and PBSA results. Graphs were generated using the numbering of EGF residues for a) PBSA and b) GBSA results. The trace for EGF is colored blue, AR is colored purple, BTC cyan, EPG brown, EPR green, HB-EGF yellow and TGF-α orange.

**Figure 7:**
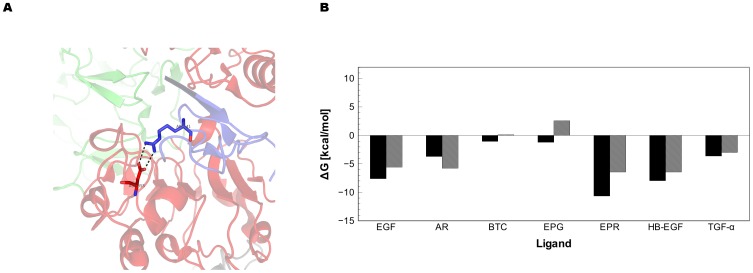
A Conserved arginine in loop 3 is important for EGFR ligand binding a) Arg41 forms a salt bridge in the EGF-EGFR x-ray structures (PDB IDs 1IVO and 3NJP) with Asp355. The energy decomposition values for Arg41 vary for each ligand. GBSA values are depicted as solid black bars and PBSA values as shaded gray bars.

The results of the residue decomposition analysis revealed several amino acids that contribute to the overall binding energy from the MM-PBSA/GBSA calculations (Figure 6). Six conversed cysteines in each ligand are necessary for constraining three loops that interact with the two ligand binding domains of EGFR. In addition to providing structural stability, we found the fourth and fifth cysteines contribute favorably to the overall binding energy by 0.00 to 3.76 kcal/mol. This observation agrees with the hydrogen bond network formed by the backbones of the fourth and fifth cysteines as well as glutamine 16 of EGFR. Another favorable energetic contribution comes from a non-conserved residue separating these two cysteine residues, providing 1.94 to −4.32 kcal/mol for six ligands. Several other conserved residues were identified to contribute to binding: a conserved aromatic residue at position 13 of EGF (−0.09 to −2.34 kcal/mol), a hydrophobic residue at position 15 (−1.20 to −3.45 kcal/mol), a non-conserved residue at position 28 (−0.10 to −4.23), two uncharged residues at positions 30(1.21 to −3.36 kcal/mol), and 38(0.48 to −4.50 kcal/mol and a conserved arginine at position 41. Previous mutational studies have shown the conserved arginine 41 (ARG41) is a requirement for EGF binding [28]. Mutation of ARG41 to any other amino acid reduces binding affinity to less than 1% of the wild type binding affinity. The energetic contribution of the conserved ARG41 varied among ligands (Figure 7). The GBSA values were all negative and ranged from −1.04 to −10.66 kcal/mol while the PBSA values varied from 2.59 to −6.24 kcal/mol.). Salt bridges between ASP355 on the ligand binding domain II and ARG41 were not observed for HB-EGF and BTC. In the case of BTC, the energetic contribution was small (<−1.03 kcal/mol). This is in contrast to HB-EGF where arginine made a significant contribution (−6.42 to −7.96 kcal/mol). While no salt bridge was detected during the course of the simulation for the conserved arginine in EPG, it did contribute favorably to the overall ΔG in the GBSA calculations (−1.21 kcal/mol) but was unfavorable for the PBSA calculation (2.59 kcal/mol). Position 32 (EGF: ASN32) also exhibited favorable energetic contributions for all ligands. Position 32 is not conserved among the members of the EGFR ligand, yet provides −2.28 to −4.32 kcal/mol for all seven EGFR ligands. While several conserved interactions contributed variable amounts of energy to the overall binding, one non-conserved position predicted to have a large impact on the binding energy similar to the contribution made by the ARG41. This position is located after the six conserved cysteine and had variable effects for each ligand and solvation method: favorable for BTC in both PBSA and GBSA calculations, favorable for EGF with GBSA (<−1.0 kcal/mol) and PBSA results and <−1.0 kcal/mol for PBSA/GBSA with AR. Both PBSA and GBSA contributions for TGF-α were unfavorable while both were favorable in the case of HB-EGF. Favorable GBSA vales for EPG and EPR occurred in this position while PBSA energies were unfavorable for the two. The range is +6.0 to −7.01 kcal/mol and appears to have a dominant effect on the ranking. These results suggest that while some conserved residues may be necessary for binding, non-conserved residues in structurally similar positions may also provide a significant contribution to the overall binding constant. It also appears that reduction of interaction energy for conserved residues affects binding affinities of the EGFR ligand family.

## Discussion

Protein-protein interactions drive biological processes [29], [30]. Ligand binding can turn on cellular signaling pathways and modulate their amplitude and duration [31]. In the case of EGFR, seven ligands are capable of binding the receptor resulting in divergent biological responses. All mature EGFR ligands share a common structure with multiple conserved residues involved in binding [11]. Binding affinities for these ligands span three orders of magnitude yet are still able to drive receptor activation in a similar manner. While the physiological and cellular effects of ligand binding have been studied extensively, the atomistic determinants that give rise to binding affinity for an EGFR ligand are still poorly understood. In this study we chose to explore these molecular interactions using molecular dynamics simulations and free energy post processing methods.

EGFR forms multiple intramolecular interactions with a ligand by binding in a clamp-like fashion on domains I and III [5], [6]. Ligand coordination is necessary for dimerization and stabilization of the complex. Following ligand binding, intracellular kinase and juxtamembrane domains undergo substantial conformational rearrangements, promoting phosphorylation of the C-terminal tail leading to recruitment of scaffold proteins [32]. Structural studies have greatly enhanced our knowledge of EGFR activation but have not been able to capture structural snapshots of binding of all EGFR ligands or a structure of the full-length receptor [33]. To address this gap in our knowledge of ligand-dependent activation of EGFR, we chose to perform molecular dynamics simulations of the dimeric state of the extracellular domain bound to each ligand. To characterize the effects of ligand binding on the conformations of the EGFR dimers, we compared structures generated during simulation runs to previously solved x-ray structures of EGFR bound to TGF-α and EGF, and the recently solved structure of another ErbB4-ligand complex [5], [6], [18]. The structure of a *Drosophila* homolog of EGFR bound to the EGF-homolog Spitz has also been determined [34].The overall conformations of the EGFR-ligand complexes show asymmetrical dimers in the presence of all seven ligands. We also observed asymmetrical dimer formation for all of our simulations, suggesting that human EGFR uses a similar mechanism as *Drosophila* EGFR, but those properties cannot be captured with static structures. [18] argues that distortion of the dimerization domain by ligand binding can not only affect the stability of the complex but also provides one possible explanation for the negative cooperativity observed in cell binding assays. The authors from this study claim that the solved structure of ErbB4 explains the lower binding affinity by confirmation of loops found in the dimerization interface. These loops are staggered in the ErbB4 complex and the EGF-EGFR x-ray structure but flush in the TGF-α, possibly to the interactions of domain IV. We found similar loop conformations in our simulations. Interestingly, EPG loops showed extreme staggering, suggesting the ligand distorts domains I and III. EPG is also a low affinity EGFR ligand. Some of the higher affinity ligands were able to form nearly flush loop conformations in the presence of domain IV in the dimer model. This leads us to a similar conclusion that [18] made: that the degree of domain II distortion may play a role in dimer stability and possibly affect the binding affinity. We do recognize, however, that we are only modeling the extracellular portion of the receptor and these conformations could be changed when adding the transmembrane and intracellular domains of EGFR.

Theoretical free energy methods allow prediction of both relative and absolute binding energies from molecular dynamics simulations [35]–[37]. The MM-PBSA/GBSA methods are efficient ways to predict binding energies for large protein-ligand and protein-protein complexes [38]–[40]. Ideally one would calculate the enthalplic and entropic contributions of a protein-ligand complex to compare with experimental binding assays. Unfortunately entropic calculations using quasi-harmonic methods are only approximate and can be computationally expensive. Forgoing inclusion of entropic terms in the binding analysis prevents determination of a total ΔG but does allow for ranking of ligands using the relative MM-PBSA/GBSA free energies. This was necessary for us as 600 frames of each EGFR-ligand simulation system were used for ΔG post-processing and contained over 200,000 atoms including solvent water molecules.

Our MM-PBSA/GBSA results provided a ranking of ligands that is close to the rankings according to previously determined binding constants [3], [16]. The PBSA results (EGF>HB-EGF>TGF-α>BTC>EPR>EPG>AR) were able to accurately rank the ligands with the exception of EPR. The original K_d_ value measured for EPR was done using the mouse homolog which contains several mutations in loop 2 and a mutation in loop 3 that have negative PBSA/GBSA decomposition energies. This could explain why the original binding affinity constant is significantly lower than our predicted binding energy. Overall, hydrophobic interactions along with electrostatic and van der Waals interactions were the energetic driving forces for binding of all seven ligands. The binding modes were similar in all cases; domains I and III formed multiple hydrogen bond networks and salt bridges with each ligand. Analysis of hydrogen bond lifetimes showed that several hydrogen bonds formed, but specific networks were not conserved across all ligands (Table 3 and Figure 5). The only hydrogen bonds formed in all cases was between the backbone of the fourth and fifth conserved cysteines and glutamine 16 of domain I. Another residue, serine 99 in domain I, was able to form hydrogen bonds with different residues on each ligand, with the exception of EPG. Another strong interaction between receptors and ligands that can be determined using simulation methods is salt bridge formation. For the case of EGFR, lysine 13 on domain I formed salt bridges with glutamates proximal to the conserved arginine. This glutamate is partially conserved among 4 ligands (EGF, AR, HB-EGF, and EPG). This partially conserved residue is replaced with hydrophobic residues in BTC, TGF- α, and EPR. TGF-α, however, is able to form a salt bridge with a glutamate occurring two positions after the arginine. Another salt bridge that may be affecting the binding affinity occurs between the conserved arginine and aspartate 355 on domain III of EGFR. This interaction is conserved in all high affinity binding ligands and also in EPR.

The PBSA results provide a roughly accurate ranking of the ligands, but do not explain the low binding energy we calculated for TGF-α. One possible explanation for this is that non-linear solvation models may overestimate the binding energy of charged atomic species. To further explore the relationship between the non-covalent interactions found in each EGFR-ligand system and an energetic contribution to the binding energy, we used energy decomposition analysis with the MM-PBSA/GBSA methods. The advantage of using decomposition analysis is it allows for electrostatic contribution to the binding energy to be determined at the amino acid residue level. This information can shed some light on the atomic determinants of binding by determining the energetic contribution for each residue in the protein and the ligand. We found several conserved residues contribute favorably to the overall binding energy. The PBSA decomposition of Glu44 in TGF-α showed this residue was contributed a significant energetic penalty to binding (4.98 kcal/mol) (Figure S4). Further analysis of the other six ligands revealed the PBSA energies for this position either contributed unfavorably or zero value to the overall binding energy. The GBSA results predicted zero or slightly negative values for six of the seven ligands. GBSA results for Glu44 of TGF-α also predicted unfavorable free energy contribution but with a value of less than 2.0 kcal/mol. The negative values predicted for this position by GBSA appear to balance out the energy values for the conserved arginine. If more favorable GBSA energy values were predicted for this Glu44 position a given ligand, the magnitude of the arginine contribution was decreased. This charge-energy compensation effect was not repeatable with PBSA energy predictions.

In summary, we have performed molecular dynamics simulations of EGFR bound to each of its endogenous ligands. This study provides for the first time, to our knowledge, a detailed view of binding of all known EGFR ligands. We observed a similar binding mode during the course of our simulations and our MM-PBSA relative free energies were in agreement with previous experimental data. GBSA results produced similar results but failed to accurately rank EPR. We acknowledge that our studies were done on only the extracellular domain and that the absence of intracellular domains may affect our binding energies due to conformational restraints. The accurate ranking of all seven ligands suggests that binding is not affected by the intracellular domains, though cooperative effects observed in full length receptors cannot be ignored. Using residue level energy decomposition analysis, we found several conserved and non-conserved residues that the contributed to the overall binding energy. We identified several conserved residues that contribute favorably to the binding energy. We were also able to identify residues that were position conserved in the structure of each ligand that contributed to our predicted binding energies. The knowledge of both sequence and structural information in regards to binding may be applied to development of therapies targeting over-expression of a particular set of EGFR ligands.

## Materials and Methods

### Structure preparation

Seven systems were constructed to model the binding of the extracellular domain of EGFR to its endogenous ligands. The initial extracellular dimer crystal structure bound to two EGF molecules (PDB id 1IVO) was used [6]. In this structure only the first three domains of EGFR were resolved; the fourth domain was not initially fit to the electron density maps. We chose to model domain IV to capture the structural features of the entire extracellular domain. To do this we used the monomeric EGFR extracellular domain bound to EGF (PDB ID 1NQL) which contains domain IV and aligned domain III with domain III with the EGFR-EGF complex. We then fused the domain IV to the C-terminal chain of the dimer structure. Recently [16] re-refined the initial x-ray data and was able to successfully fit domain IV to the density maps (PDB id 3NJP). Our model showed an overall similar conformation to this structure with a root mean squared deviation of 2.89 angstroms.

X-ray structures have been solved for complexes with only two EGFR ligands bound: EGFR and TGF-α. Three ligand structures have been solved using NMR (AR, BTC, and EPR) and one x-ray bound to the diphtheria toxin (HB-EGF; see table 1). The structure of EPG, the last discovered of the EGFR ligands, remains unsolved. In the absence of EPG structural data, we used a previously determined homology model of EPG from the SWISS repository [41], [42]. The EPG model was predicted using the GROMOS96 force field and was subject to 200 cycles of steepest descent energy minimization followed by 300 cycles of conjugate gradient minimization. Each ligand structure was then docked to the EGFR dimer by alignment to the EGF molecule. To remove steric overlap, we used the energy minimization function in the UCSF Chimera visualization software [17]. Rigid body docking procedures can lead to errors depending on the type of docking search performed. Since we chose to manually dock our ligands, we checked one complex against available structural data. One such structure is that of TGF- α bound to an EGFR dimer. After MD optimization of our initial docking, we performed cluster analysis and compared the binding poses of the highest 10 populated states to the x-ray structure of TGF- α bound to EGFR. The binding domains of EGFR adopted a similar confirmation to the x-ray structure and the backbone of TGF- α was in a similar position with respect to its bound confirmation in the x-ray structure. Structural representations were visualized in Pymol [43].

### Molecular dynamics

All simulations and system equilibrations were performed using Amber 11 software. The leap module of AMBER11 was used to create parameter and topology files for the MD simulations using the AMBERff03.r1 force field [44]. Hydrogen atoms were added and ionizable residues were set to predicted protonation states at pH 7.0. Na^+^ counterions were added to each system to achieve neutrality. TIP3 water molecules were added with a minimum spacing of 10.0 Å from the box edges to the protein molecule. Each system contained >200 k atoms. Energy minimization on each system was performed in a two-step process. First the protein atoms were restrained and the water molecules were allowed to relax over 1000 steps. The entire system was then subjected to energy minimization using the steepest descent method for the first 1000 steps, followed by the full conjugate gradient method for an additional 2400 steps. Each system was then heated to 300 K for 100 ps followed by a 50 ps constant pressure simulation to adjust the density to 1 g/mL. An additional 500 ps simulation was run prior to production simulations to allow for further temperature and pressure equilibration. Production runs were performed using a canonical ensemble (NVT) scheme. Langevin dynamics with a collision frequency of 2.0 were used for temperature regulation, the SHAKE algorithm was used for all hydrogen atoms, and the particle mesh Ewald (PME) method was employed to treat long-range electrostatics and van der Waals forces (cutoff of 8 Å) with an integration step of 2.0 fs. All Amber equilibration and production runs were performed using dual precision. All production simulations were repeated in triplicate and extended to 25.0 ns. Visualization of trajectories was performed in VMD [45].

### MMPBSA/MMGBSA Calculations

The binding energies between EGFR and each ligand were calculated using the MM-PBSA/MM-GBSA method in Amber11 [46], [47]. The MMPBSA method calculates a binding free energy by the free energies of solvation for the complex (ΔG_complex_), protein (ΔG_protein_) and ligand (ΔG_ligand_):
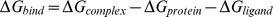
Each term is calculated by determining the enthalpic energy of the solute using molecular mechanics (E_MM_), the polar solvation free energy (ΔG_solv_), the nonpolar solvation free energy (ΔG_np_) and the entropic contribution (ΔS):
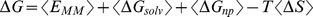
The enthalpic term is taken as the average over the molecular mechanics force field terms for the solute. The solute vibrational entropy is estimated using either normal mode analysis or quasi-harmonic approximation. ΔG_solv_ is solved using the Poisson Boltzmann (PB) equation. The nonpolar term ΔG_np_ is solved using the Generalized Born (GB) method and is assumed to be proportional to the SASA [48]:
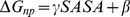
Where γ is the surface tension, set to 0.0072 kcal/Å^2^. β is an offset value used to correct for the nonpolar contribution to the solvation free energy term and is dependent on the GB model used [49].

For each EGFR-ligand system, MM-PBSA and MM-GBSA calculations were performed using 600 snapshots over the last 6.0 ns of the simulation with 100 ps intervals. All energy values, including decomposition values, represent at least two independent MD simulation runs for each ligand-protein complex. All calculations were performed with the MMPBSA.py.MPI module in Amber 11 with an ionic strength equal to 0.1 M. For MM-PBSA calculations the PB equation is solved numerically by the PBSA program included with AmberTools. The hydrophobic contribution is approximated by the LCPO method implemented within sander [50]. For MM-GBSA calculations the Hawkins, Cramer, Truhlar pairwise GB model was used with parameters described by Tsui and Case [51].

### Binding free energy decomposition

To determine the energetic contribution of an individual residue to ΔG, *in silico* alanine mutagenesis is usually performed, yielding a ΔΔG_ala_. A major drawback to this method is that mutations in macromolecular structures may cause perturbations that transcend the binding interface [24]. To circumvent this problem a GB model can be implemented to calculate the electrostatic contribution to ΔG_solv_ [52], [53]. The GB model is defined as:
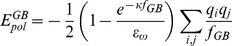
Where q_i_ and q_j_ are atomic partial charges, κ is the Debye-Huckel screening parameter and ε_ω_ is the solvent dielectric constant, which is set to 80.0 for water. *f_GB_* is defined as:
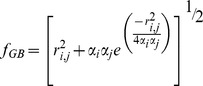
Where r_i,j_ is the distance between the ith and jth atom, α_i_ and α_j_ are the effective Born radii.

The ith atom contribution to the electrostatic free energy is obtained by solving:
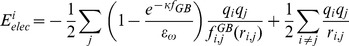
For each EGFR-ligand system the energy decomposition analysis was done using the GB and PB solvation models. The analysis was performed on the ligand and the receptor using 600 snapshots over the last 6.0 ns of the simulation with 100 ps intervals.

### Hydrogen bond and salt bridge analysis

Hydrogen bonding analysis was performed in the Hydrogen Bond module of VMD [45]. We used a distance cut off of 3.2 Å and a maximum angle of 30 degrees between the donor and acceptor heavy atoms. Using the last 6 ns of each simulation, we recorded any unique hydrogen bonds with a lifetime of ≥10% of the simulation window. The Salt Bridges program in VMD was used to determine any salt bridges between EGFR and a given ligand. For this method we used an oxygen-nitrogen distance cutoff of 3.4 Å [45].

## Supporting Information

Figure S1 **SASA fluctuations for each EGFR-ligand complex.** The trace for EGF is colored blue, AR is colored purple, BTC cyan, EPG brown, EPR green, HB-EGF yellow and TGF-α orange.
(TIF)

Figure S2 **Modeling domain IV for EGFR dimers.** a-b) The x-ray structure of the EGF-EGFR dimer containing the first three domains of EGFR(colored blue) and the monomeric EGFR-EGF complex(colored magenta) containing domains I–IV were aligned using domain III as a reference.
(TIF)

Figure S3 **Dimerization domain interface of EGFR-ligand structures.** A) Structural alignment of EGFR-EGF model (colored cyan) and TGF-α-EGFR x-ray structure (colored magenta) (PDB ID 1MOX) complexes. B) Top down view of the dimerization domains.
(TIF)

Figure S4 **Decomposition values for position for 43.** GBSA values are depicted as solid black bars and PBSA values as shaded gray bars.
(TIF)

Table S1 **Free energy results for MM-GBSA calculations of last 6.0 ns of each ligand-protein complex.**
(DOC)
