# Supplementary figures and images for: Molecular Determinants of Epidermal Growth Factor Binding: A Molecular Dynamics Study
Source: PLoS One. 2013 Jan 24;8(1):e54136. doi: 10.1371/journal.pone.0054136 (PMC3554757; doi:10.1371/journal.pone.0054136)

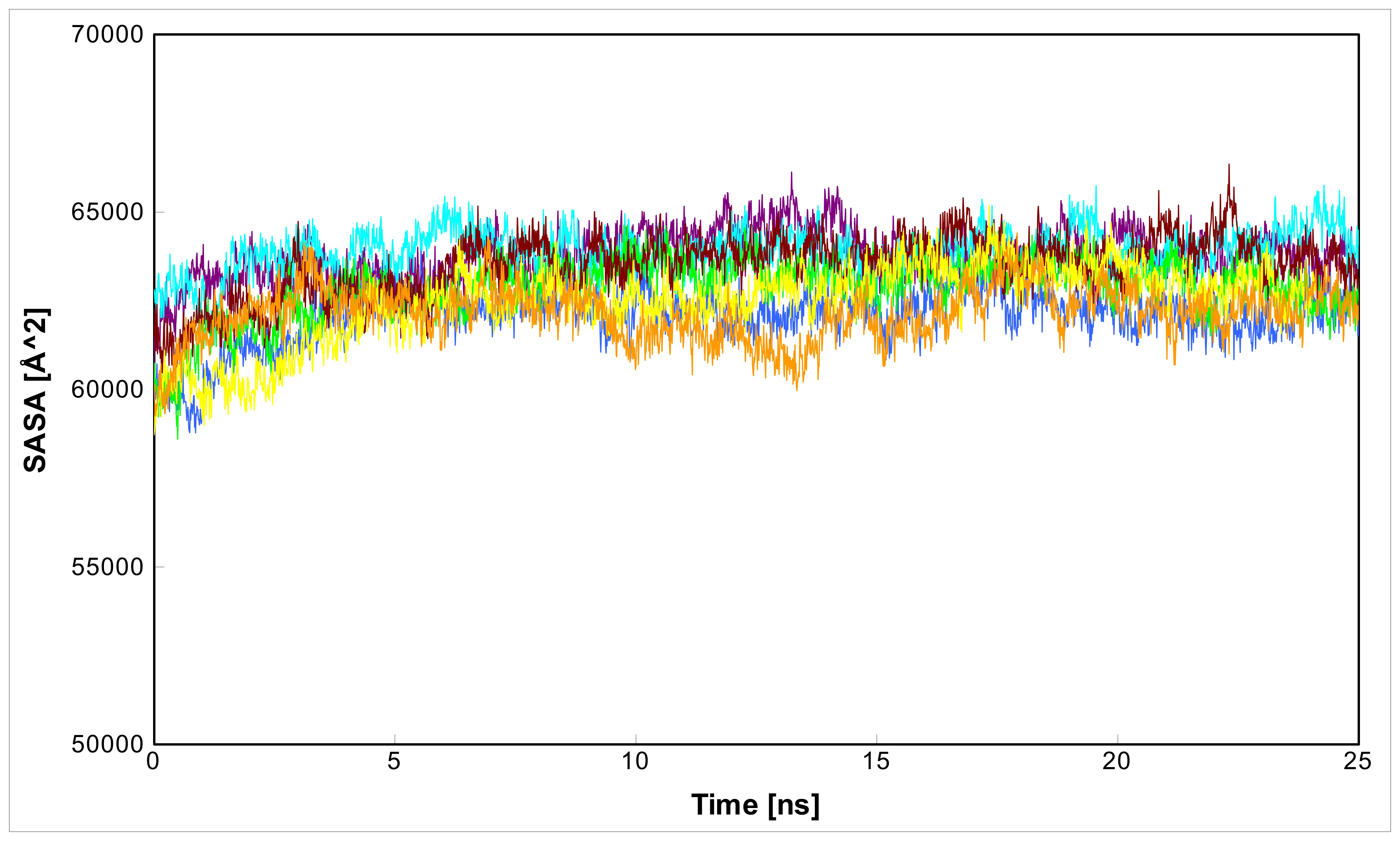

Supplement: Figure S1 — SASA fluctuations for each EGFR-ligand complex. The trace for EGF is colored blue, AR is colored purple, BTC cyan, EPG brown, EPR green, HB-EGF yellow and TGF-α orange. (TIF) [file pone.0054136.s001.tif]

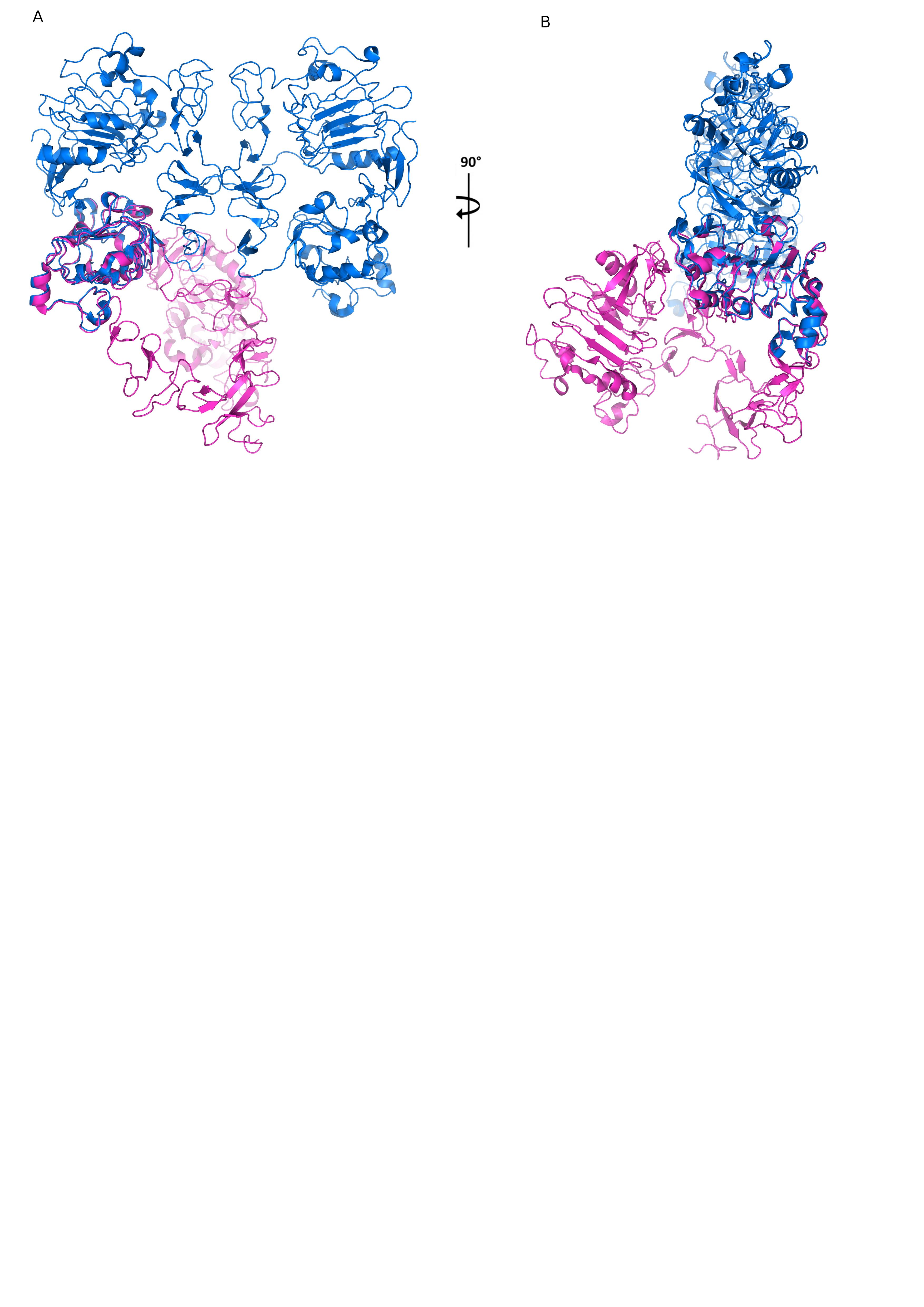

Supplement: Figure S2 — Modeling domain IV for EGFR dimers. a-b) The x-ray structure of the EGF-EGFR dimer containing the first three domains of EGFR(colored blue) and the monomeric EGFR-EGF complex(colored magenta) containing domains I–IV were aligned using domain III as a reference. (TIF) [file pone.0054136.s002.tif]

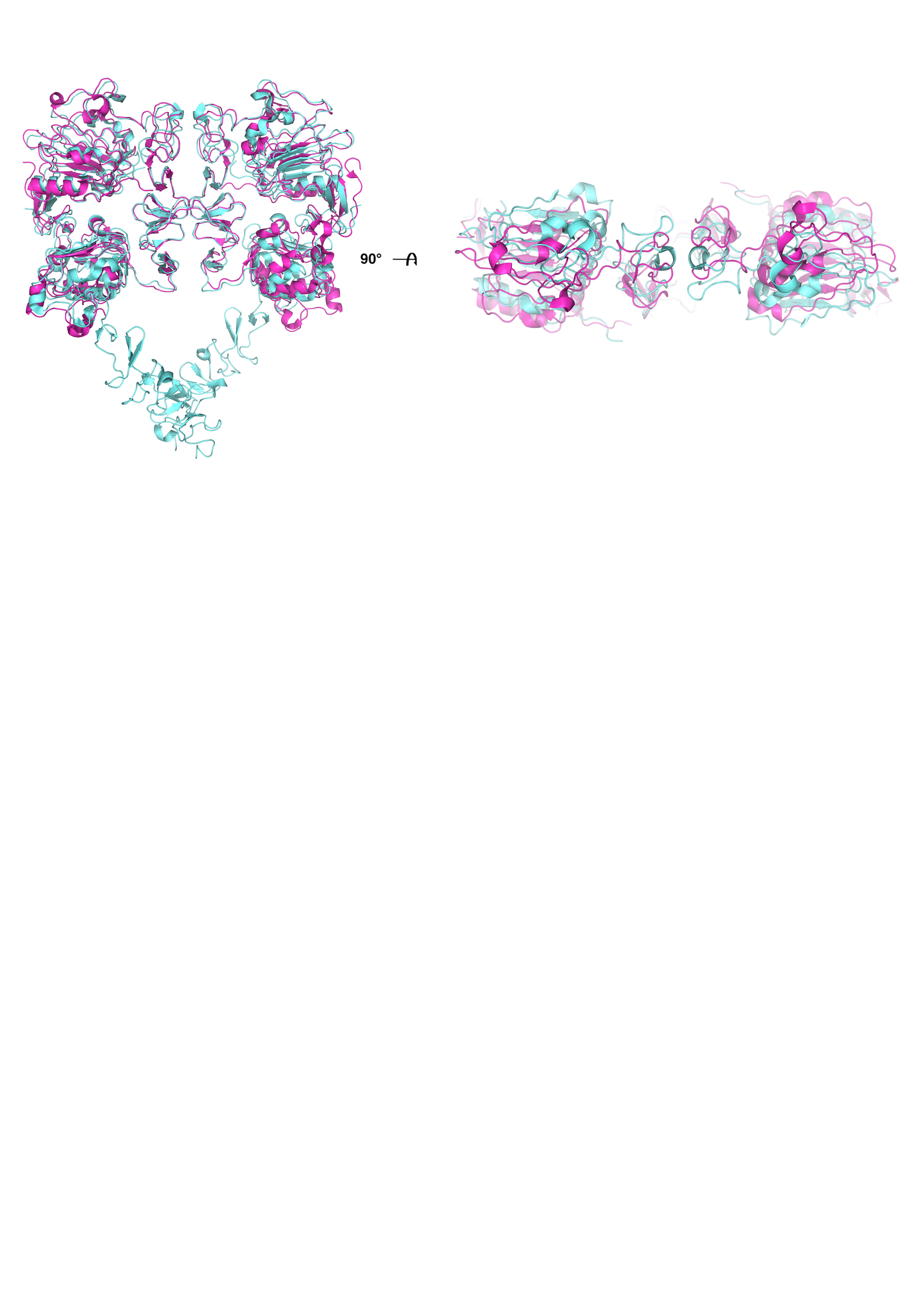

Supplement: Figure S3 — Dimerization domain interface of EGFR-ligand structures. A) Structural alignment of EGFR-EGF model (colored cyan) and TGF-α-EGFR x-ray structure (colored magenta) (PDB ID 1MOX) complexes. B) Top down view of the dimerization domains. (TIF) [file pone.0054136.s003.tif]

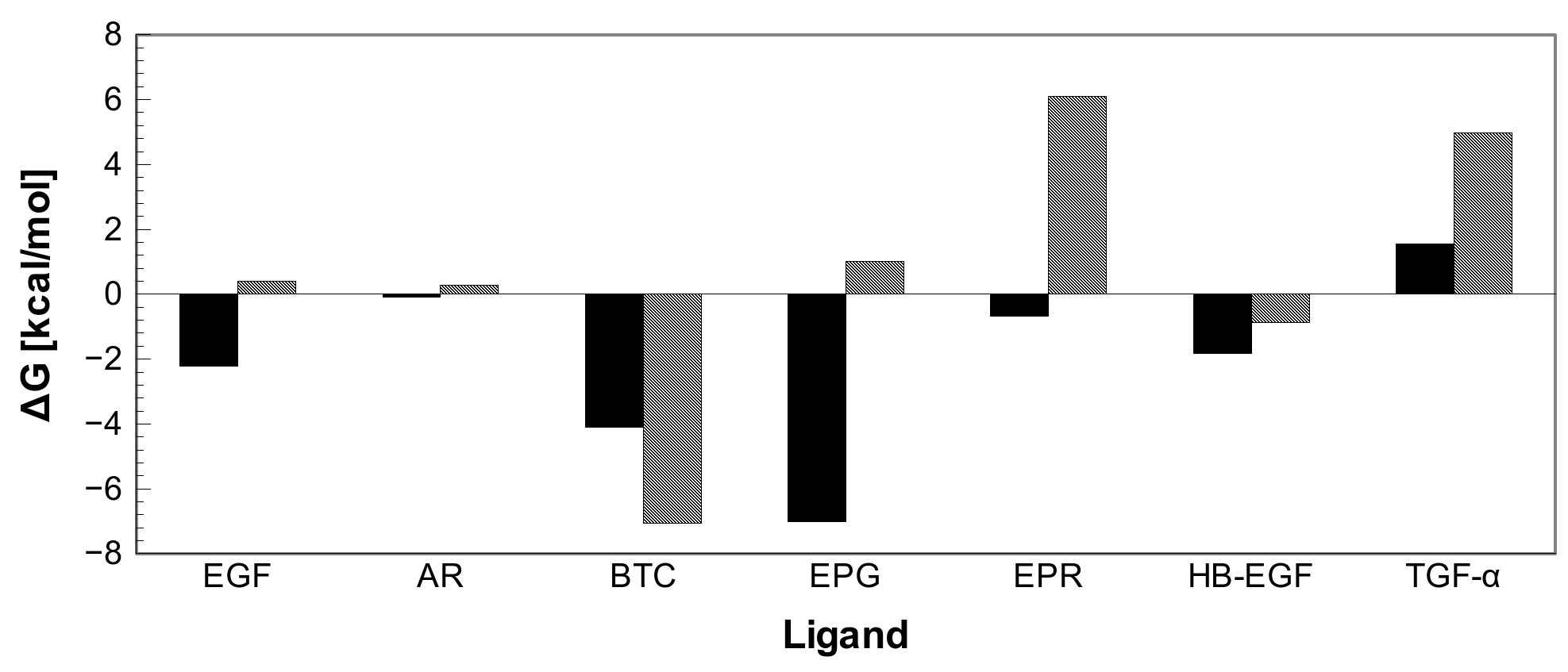

Supplement: Figure S4 — Decomposition values for position for 43. GBSA values are depicted as solid black bars and PBSA values as shaded gray bars. (TIF) [file pone.0054136.s004.tif]
