# Supplementary material for: Molecular Determinants of Epidermal Growth Factor Binding: A Molecular Dynamics Study
Source: PLoS One. 2013 Jan 24;8(1):e54136. doi: 10.1371/journal.pone.0054136 (PMC3554757; doi:10.1371/journal.pone.0054136)
Supplement: Table S1 — Free energy results for MM-GBSA calculations of last 6.0 ns of each ligand-protein complex. (DOC) [file pone.0054136.s005.doc]

**Supplementary Table 1**. Free energy results from MM-GBSA.

| Ligand | ΔEele^*^ | ΔEvdw^**^ | ΔGGB^***^ | ΔGSA^****^ | ΔGMMGBSA |
| --- | --- | --- | --- | --- | --- |
| EGF | -176.5(8.30) | -148.12(10.48) | 212.06(10.79) | -23.97(1.20) | -134.91(9.12) |
| HB-EGF | -365.82(2.65) | -115.22(8.08) | 386.90(5.88) | -18.38(0.78) | -111.45(4.57) |
| TGF-α | -146.22(55.20) | - 132.43(2.40) | 194.62(41.46) | -21.24(0.14) | -115.27(1.35) |
| BTC | -425.56(66.03) | -115.78(6.69) | 454.55(52.82) | -18.71(1.11) | -109.23(4.00) |
| EPG | -121.95(13.23) | -91.51(11.99) | 150.58(12.79) | -15.69(0.41) | -78.47(10.52) |
| EPR | -211.75(34.05) | -117.49(13.06) | 239.04(27.39) | -19.20(1.63) | -108.80(15.1) |
| AR | 207.08(0.87) | -100.29(1.13) | 238.13(0.41) | -15.02(0.88) | -85.60(0.20) |
| All units are given in kcal/mol. The standard state is taken to be 1 M.  * : ΔEele: coulombic energy.  **: ΔEvdw: van der Waals energy.  ***: ΔGGB: Generalized Borne polar solvation energy  ****: ΔGSA :non-polar solvation energy  Standard Errors of corresponding values are given in parentheses. | | | | | |
